# Factors influencing adolescents’ decision-making about COVID-19 vaccination: a systematic review with qualitative synthesis

**DOI:** 10.3389/fpubh.2025.1563677

**Published:** 2025-05-14

**Authors:** Nayara Moreira da Cunha, Sofia Tzirita, Elisa Gobbo, Sibylle Herzig van Wees

**Affiliations:** Department of Global Public Health, Karolinska Institutet, Stockholm, Sweden

**Keywords:** COVID-19, COVID-19 vaccines, vaccination hesitancy, adolescent, systematic review, qualitative synthesis

## Abstract

**Introduction:**

Attitudes towards vaccination are influenced by a broad range of factors, yet little is known about the drivers shaping adolescents’ vaccination beliefs. The aim of this study was to qualitatively explore the factors influencing adolescents’ individual decision-making towards COVID-19 vaccination.

**Methods:**

A systematic review was conducted using Medline, Web of Science, Sociological Abstracts, and Publicly Available Content Database. Studies on attitudes, beliefs, and perceptions of adolescents regarding COVID-19 vaccines were included. The JBI Critical Appraisal Checklist was used for quality assessment, followed by thematic synthesis of the included studies.

**Results:**

In total, 13 studies were included, revealing 5 key themes: (1) Limited vaccine literacy influences adolescents’ attitudes towards COVID-19 vaccines; (2) Family, peers, and community strongly influence adolescents’ COVID-19 vaccine decision-making; (3) Different levels of trust in vaccine providers and governments influence adolescents’ attitudes towards COVID-19 vaccines; (4) Desire to go back to normality influences adolescents’ COVID-19 vaccine attitudes towards vaccine acceptancy; (5) Autonomy influences adolescents’ COVID-19 vaccine decision-making.

**Discussion:**

The review findings suggest that vaccine acceptance among adolescents could be improved through tailored and accessible vaccine literacy messaging, addressing structural mistrust, and empowering adolescents to make autonomous health decisions that take into account diverse contexts and populations.

**Systematic review registration:**

https://www.crd.york.ac.uk/PROSPERO/view/CRD42024512197, identifier CRD42024512197.

## Background

Vaccination is a cornerstone of public health and among the most cost-effective public health interventions available today (1, 2). Vaccination is the act of introducing a vaccine to acquire protection by stimulating the body’s natural defences against germs and diseases (3–5). The importance of vaccination was demonstrated recently by the critical role vaccines played in mitigating the COVID-19 pandemic. During this time, the COVID-19 Vaccines Global Access (COVAX) initiative expedited vaccine development while upholding rigorous standards for safety, efficacy, and ethical integrity (6, 7). As a result, COVID-19 vaccines saved approximately 14.4 million lives worldwide in 2021, contributing to a 63% reduction in global COVID-19-related deaths in the first year of vaccination (8, 9). Despite these achievements, a recent systematic review from 2023 estimated a global COVID-19 vaccine hesitancy rate of 29.72%, largely driven by low confidence and high levels of complacency (10).

Vaccine hesitancy is not a new phenomenon (11). It has been recognized by the World Health Organisation (WHO) as one of the top 10 threats to global public health (12). The Strategic Advisory Group of Experts on Immunization (SAGE) defines vaccine hesitancy as a “delay in acceptance or refusal of vaccination despite the availability of vaccination services” (13), encompassing a continuum of attitudes (13, 14). Vaccine hesitancy has been described as a complex phenomenon, varying by time, place, and vaccine. It is influenced by factors such as complacency, convenience, and confidence, known as the WHO’s ‘3Cs’ model (13). An important aspect of vaccine confidence is trust, which is based on relationships between individuals or between individuals and a system, where one party willingly assumes a position of vulnerability, relying on the competence and good intentions of the other in order to simplify decision-making (15).

Additionally, vaccine hesitancy must be understood within its social, historical, and political context. In each setting, public health policies, healthcare workers’ (HCWs) recommendations, and media communication intersect to influence attitudes, with the level of trust playing an important role in this dynamic (14). For COVID-19 vaccines, primary global drivers of hesitancy have included a low perceived risk of infection and severity, limited institutional trust, and concerns regarding vaccine safety (16, 17). However, in different population subgroups, there are specific determinants that affect hesitancy.

Adolescents are a particularly important, yet understudied, subgroup for vaccination efforts, with distinct characteristics that set them apart from other age groups (18). For the purpose of this research, we have defined adolescence as individuals from ages 10 to 19 in the phase between childhood and adulthood, based on the WHO definition (19–21). Adolescence is a transitional phase, especially for decisional rights (22). Within this age range, individuals gain both legal autonomy and capacity for informed consent, but the exact age of consent varies by country (19, 21). This leads to a new navigation of the balance between authority figures’ influence and individual autonomy in informed health-related decision-making (23). The process of decision-making involves evaluating outcome alternatives through analysing gathered information and further selecting a course of action (24, 25). Yet, even in instances with legal consent, adolescents’ decision-making regarding vaccination often involves parents and HCWs.

Parental and HCW hesitancy, rooted in concerns about vaccine safety and related misinformation, institutional mistrust, and perceived rights infringements, especially regarding COVID-19, can lead to lower vaccination rates among adolescents (23, 26–32). In addition, a quantitative review on adolescent vaccine hesitancy identified individual key barriers to vaccination, such as low awareness, perceived low efficacy, and safety concerns, while facilitators included knowledge, perceived efficacy, information access through family and school, and autonomy in decision-making (18).

Given the public health importance of vaccination and risks posed by new variants and outbreaks, this study aims to enhance understanding of the factors influencing adolescents’ attitudes towards COVID-19 vaccination by synthesising qualitative studies on the topic. By exploring the nuanced factors influencing their perceptions, our findings can provide valuable insights to help develop tools to adequately measure and monitor adolescent vaccine confidence and identify strategies to address adolescents’ vaccine hesitancy.

## Methods

The systematic review was based on the Preferred Reporting Items for Systematic Reviews and Meta-Analysis for Protocols 2015 (PRISMA-P 2015) guidelines (33). The guiding research question is ‘What are the factors, as described by adolescents, that influence their individual decision-making about COVID-19 vaccination?’. The PEO (Population, Exposure, and Outcome) tool was used in order to focus on the systematic analysis of qualitative research, in which associations between exposure and health outcomes are explored and can further guide health policies (34, 35). Then the research question was developed based on (P) Adolescents aged 10–17 years-old; (E) Factors such as adolescents’ knowledge, perceptions, and views about vaccination and COVID-19 vaccines; (O) Attitudes towards COVID-19 vaccines and vaccination decision-making (36).

The systematic review study protocol is registered in PROSPERO with the identification number CRD42024512197 (37). The original research question submitted in the PROSPERO protocol was: ‘What is the available evidence on adolescents’ perspectives, views, and attitudes about vaccines and vaccination in relation to this population’s COVID-19 vaccine hesitancy status?’ However, during the review, it was revised to better focus on the specific factors and attitudes influencing adolescents’ decisions, ensuring a more targeted and relevant analysis. To ensure comprehensive reporting, the authors followed both the PRISMA-P framework for review reporting (33) and the Enhancing Transparency in Reporting the Synthesis of Qualitative Research (ENTREQ) checklists (38).

### Data sources and searches

The last search was performed on 23 February 2024 in the following databases: Medline, Web of Science, Sociological Abstracts and Publicly Available Content Database (in collaboration with librarians at the Karolinska Institutet University Library). The search strategy was first developed in Medline (Ovid). For each search concept, Medical Subject Headings and free text terms were identified in Medline. The search was then translated, in part using Polyglot Search Translator (39), to efficiently translate search strategies across multiple databases, converting search strategies from Ovid MEDLINE into formats compatible with databases such as Cochrane Library, Embase (via Elsevier and Ovid), Web of Science, and others. Key terms for the search included vaccine, immunization, attitude, belief, trust, adolescent, young adult, and youth. No language restriction was applied, and articles older than 2020 were not reviewed. This decision was taken considering that COVID-19 emerged on the global scene in late December 2019. Thus, a comprehensive understanding of disease characteristics, burden, knowledge, and vaccine availability was not available prior to 2020.

De-duplication was done using the method described by Bramer et al. (40). One final, extra step was added to compare DOIs. A manual snowball search was applied to check references and citations of eligible studies from the database searches. The full search strategies for all databases are available in Appendix 1.

### Study selection

NMC and EG independently screened the articles by reviewing titles and abstracts, followed by full-text assessments, based on the established inclusion and exclusion criteria. The inclusion criteria were: (1) qualitative or mixed-methods studies; (2) studies in English, Portuguese or Swedish; (3) study participants—adolescents aged 10 to 17 years old (studies that presented data from younger or older individuals were also included as long as the data extracted could be identified as from a person within 10 to 17 years old and/or the majority of subjects were within 10–19); (4) studies focusing on vaccine hesitancy, acceptance, and/or refusal of adolescents regarding COVID-19 vaccines and vaccination; and (5) studies focusing on knowledge, beliefs, views, perceptions and attitudes of adolescents regarding COVID-19 vaccines and vaccination. The exclusion criteria were: (1) studies focusing on parents and/or guardians’ knowledge, beliefs, views, perceptions, and attitudes regarding vaccination for their children; (2) studies focusing on adolescents’ knowledge, attitudes, and perceptions about general vaccination or other non-COVID-19 vaccines; and (3) studies focusing on HCWs’ knowledge, attitudes, and perceptions about COVID-19 vaccine hesitancy in adolescents. The process of revision and selection of articles was performed with the assistance of the *Rayyan.ai* tool (41). Conflicts were resolved among the team.

### Data extraction

Data were extracted by NMC according to a predetermined excel data extraction table, including title, authors, year of publication, aim/objective, country, data collection period, study design, data collection method, sample size/participants characteristics, full results section (themes identified, codes identified, and quotes used). The extracted data were then reviewed by EG.

### Quality assessment

Assessment of risk of bias on the included articles was performed by NMC and EG utilizing the Joanna Briggs Institute (JBI) Critical Appraisal Checklist for Qualitative Research (42). This tool was used to evaluate qualitative studies in a well-structured manner, allowing for a systematic and transparent assessment.

### Data synthesis

A qualitative synthesis strategy was used to systematically evaluate and synthesize the results of the included qualitative or mixed-method studies (43). The process of qualitative synthesis included an analysis using summarization, descriptive statistics, and thematic synthesis of the selected articles. Prior to synthesis, a standardized data extraction sheet was developed that included: title, authors, year of publication, aim/objective, country, data collection period, study design, data collection method, sample size/participants characteristics, results—themes identified, codes identified, quotes used.

The extracted qualitative results were interpreted and integrated to identify recurring patterns and generate themes. An inductive thematic synthesis was performed by NMC in accordance with Thomas and Harden’s qualitative synthesis strategy, which is a strategy that was developed to address research questions related to the acceptability of interventions (43, 44). The analysis strategy evaluates barriers and facilitators of interventions; thus, it was used here to understand adolescents’ perceptions of vaccination. This synthesis helped us draw broader conclusions and gain a deeper understanding of adolescents’ experiences and expressed needs. An initial inductive line-by-line coding of the primary study findings was conducted, followed by organizing these codes into descriptive themes, which led to the development of analytical themes. To assist in the coding process, *Atlas.ti* software and *Excel* were utilized (45). In the final stages of coding, regular discussions of the themes among NMC, EG and SHvW were performed.

### Ethical considerations

This study is an evaluation of published, publicly accessible studies and hence, no formal ethical approval was required according to the Swedish Ethics regulations.

## Results

**Figure 1:**
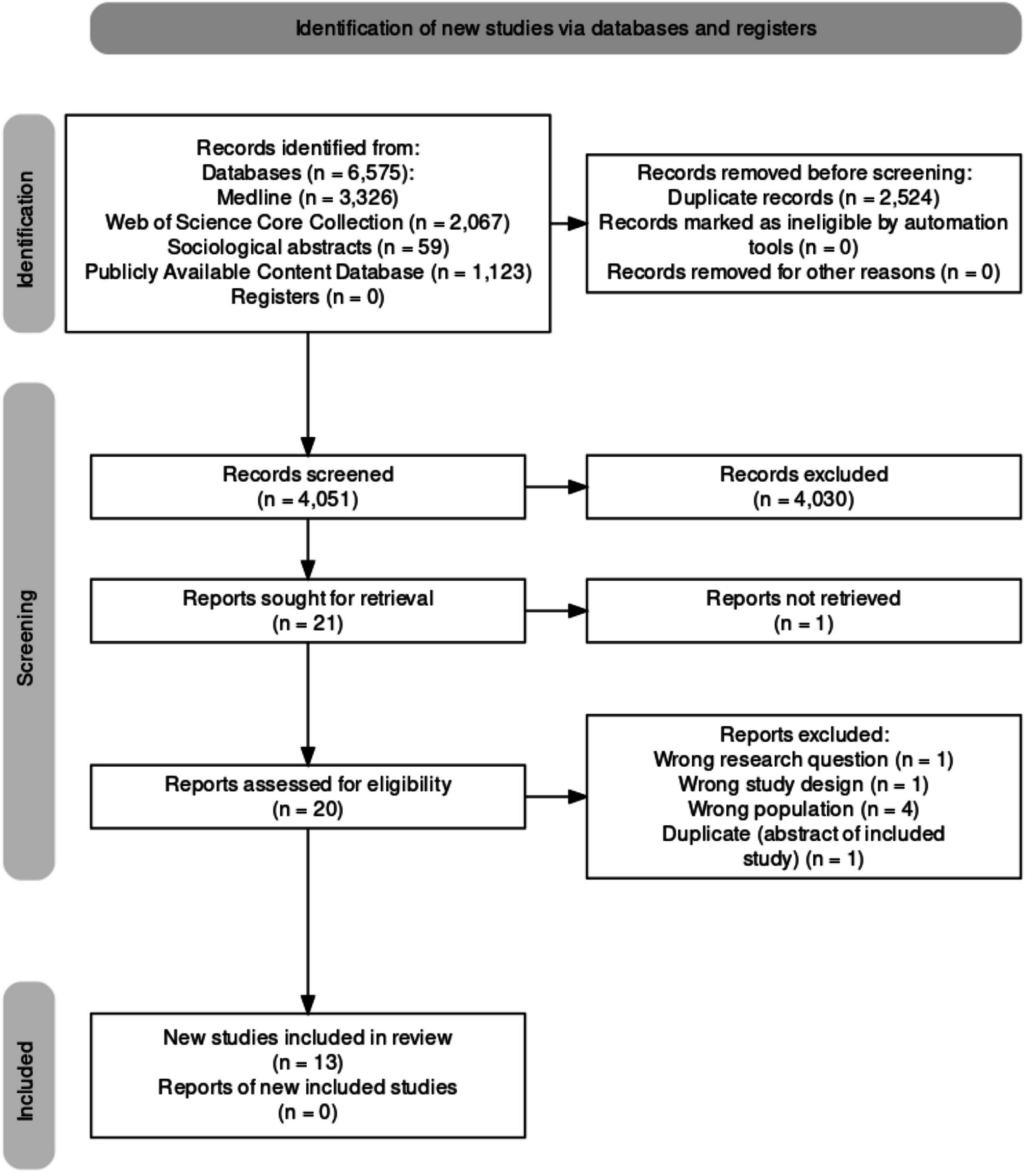
PRISMA 2020 flowchart of study selection process (71).

A total of 6,575 records were initially identified and out of the 4,051 records screened after deduplication, 13 studies (46–58) were eligible for inclusion. The study selection process is illustrated in the PRISMA 2020 flowchart in Figure 1.

### Characteristics of the included studies

Nine of the thirteen studies utilized interviews as their primary data collection method (47–49, 51–53, 55–57), two conducted focus groups (50, 54), and two used open-ended responses for qualitative analysis (46, 58). The studies represented a range of countries: six from the United States (US) (51, 54, 56–58), two from Canada (48, 49), and one each from South Africa (55), Ethiopia (53), Uganda (47), England (52), Sweden (46), Brazil (50), China (50), the Democratic Republic of Congo (50), Indonesia (50), Belgium (50), and Malawi (50). Additionally, seven studies focused on adolescent populations from vulnerable backgrounds (47–49, 51, 54, 57, 58), such as those experiencing homelessness, refugee status, or belonging to historically marginalized communities. The articles are all summarized in Table 1.

**Table 1:** Summary of key information from included articles.

Title, first author, and year of publication	Country and data collection period	Study design and data collection method	Aim / objectives	Results (themes, categories, codes)
(1) COVID-19 vaccine hesitancy and its associated factors among adolescents, Alemu et al. (2023)	**Ethiopia** *June 2022 to July 2022*	Mixed-methods *In-depth interviews*	“To assess the magnitude of COVID-19 vaccine hesitancy and its associated factors among adolescents in Seka Chekorsa town, Jimma, Ethiopia.”	NA (not described as themes, categories or codes)
	**Participants’ characteristics**			
	School adolescents in the age group of 12–17 years *Qualitative sample not specified*			
(2) COVID-19 vaccine attitudes among youth experiencing homelessness: a qualitative analysis with opportunities for action, Balma et al. (2023)	**USA** *October to November 2021*	Qualitative *Focus groups*	“To describe youth perspectives that influence COVID-19 vaccine confidence and uptake, and identify youth-driven strategies to guide public health efforts to improve vaccine confidence and access.”	(1) “Historical harms and mistrust of systems”; (2) “Access to reliable information”; (3) “Basic needs as youth’s priority”; (4) “Personal health influence”; (5) “Barriers to health care”; (6) “Fear or uncertainty of vaccine”; (7) “Sense of bodily autonomy”; (8) “Community influence”
	**Participants’ characteristics**			
	Youth aged 12–24 years-old experiencing homelessness *N = 20*			
(3) “It does not cure, but it protects”: COVID-19 vaccines through the eyes of children and their parents, Groenewald et al. (2023)	**South Africa** *January to December 2021*	Mixed-methods *Interviews*	“To explore the intergenerational influence that parents’ perspectives had on children’s vaccine acceptability and the role that vaccine literacy, or lack thereof, played in vaccine decision making.”	(1) “Children’s Perspectives on COVID-19 Vaccines”; (2) “Intergenerational Influence of COVID-19 Perspectives”
	**Participants’ characteristics**			
	Dyads of parents and/or caregivers, and adolescents younger than 18. *N = 29 (16 adolescents)*			
(4) Multi-method findings on COVID-19 vaccine acceptability among urban refugee adolescents and youth in Kampala, Uganda, Logie et al. (2023)	**Uganda** *March 2021*	Mixed-methods *Survey and semi-structured in-depth interviews*	“To investigate COVID-19 vaccine acceptability among young urban refugees ages 16–24 living in Kampala, Uganda”	(1) “Barriers to vaccine acceptability”; (2) “Facilitators of vaccine acceptability”
	**Participants’ characteristics**			
	Refugees aged 16–24 years *N = 24*			
(5) Decision-making about COVID-19 vaccines among health care workers and their adolescent children, Mansfield et al. (2023)	**USA** *November 2021 to December 2021*	Qualitative *Semi-structured interviews*	“To explore how vaccinated health care workers approach decision-making for COVID-19 vaccination with their adolescent children.”	(1) “Family Anticipation and Hesitation about the Approval of COVID-19 Vaccines for Adolescents”; (2) “Parents’ choice or adolescents’ choice: The decision maker for adolescent COVID-19 vaccination”; (3) “Leveraging one’s own vaccination status to encourage others to get vaccinated”
	**Participants’ characteristics**			
	Adolescents children aged 12 to 17 of physicians, nurses and medical staff practicing at KPSC (Kaiser Permanente Southern California) *N = 17*			
(6) Perspectives on COVID-19 vaccination and vaccine passports in a diverse urban adolescent population: a youth participatory mixed methods study, Mckinnon et al. (2023)	**Canada** *January to March 2022*	Mixed-Methods *Survey and semi-structured interviews*	“To explore determinants of COVID-19 vaccination, attitudes and perceptions underlying vaccine-related decision-making, and opinions about vaccine passports among adolescents within this unique sociocultural and policy context.”	(1) “Being misinformed contributes to COVID-19 vaccine hesitancy among nonvaccinated adolescents”; (2) “Nonvaccinated adolescents value autonomy in making decisions about COVID-19 vaccination”; (3) “Vaccine mandates consistently described as unfair but effective”
	**Participants’ characteristics**			
	14–17-year-old who were unvaccinated against COVID-19 *N = 19*			
(7) Attitudes toward COVID-19 vaccine among pediatric patients with sickle cell disease and their caregivers, Persaud et al. (2023)	**USA** *May 2021 to February 2022*	Mixed-methods *Survey*	“To identify strategies to address vaccine hesitancy, we sought to broadly identify attitudes and barriers among unvaccinated adolescents and pediatric SCD caregivers.”	(1) “Lack of personal utility (benefit) from vaccination or not ready”; (2) “Mistrust, Misinformation, Rushed”; (3) “Unanswered medical questions”
	**Participants’ characteristics**			
	Adolescents aged 13 to 18 years-old with sickle cell disease. *N = 49*			
(8) Family, community, institutional and policy factors on COVID-19 vaccine perceptions among urban poor adolescents in seven countries: qualitative cross-site analysis, Ramaiya et al. (2023)	**Belgium**; **Brazil**; **China**; **Democratic Republic of Congo**; **Indonesia**; **Malawi** and **USA** *March 2021 to April 2022*	Qualitative *Focus groups*	“To qualitatively explore these factors (family, community, institutional and policy factors) and how they shape adolescents’ perspectives on COVID-19 vaccines across seven countries”	(1) “Family factors”; (2) “Community factors”; (3) “Institutional factors”; (4) “Policy factors”
	**Participants’ characteristics**			
	Adolescents aged 13–18 years. *Belgium (N = 22); Brazil (N = 33); China (N = 40); DRC (N = 30); Indonesia (N = 66); Malawi (N = 34); USA (N = 12)*			
(9) Examining COVID-19 vaccine uptake and attitudes among 2SLGBTQ + youth experiencing homelessness, Abramovich et al. (2022)	**Canada** *January 2021 to June 2021*	Mixed-methods *Survey and interviews*	“To explore this group’s (2SLGCT+) COVID-19 vaccine attitudes, and facilitators and barriers impacting vaccine uptake.”	(1) “Mistrust in the healthcare system”; (2) “Lack of targeted vaccine‑related information”; (3) “Vaccine side effects”; (4) “Accessibility”
	**Participants’ characteristics**			
	2SLGBTQ + youth aged up to 29 years-old at risk of, and experiencing, homelessness *N = 32*			
(10) COVID-19 vaccine sentiments among African American or black adolescents in rural Alabama, Budhwani et al. (2021)	**USA** *May 2021*	Qualitative *In-depth interviews*	“To ascertain sentiments toward COVID-19 vaccination among rural AAB adolescents.”	(1) “Influence of community leaders and older family members”; (2) “Fear of side effects and misinformation”; (3) “Institutional distrust”
	**Participants’ characteristics**			
	African American or black adolescents aged 15 to 17 years-old *N = 28*			
(11) Experiences of the coronavirus disease-19 (COVID-19) pandemic from the perspectives of young people: rapid qualitative study, Fisher et al. (2021)	**England** *June 2020*	Rapid Qualitative study *Semi-structured interviews*	“To examine the experiences of the COVID-19 pandemic from the perspectives of young people.” “The specific objectives are to: (1) explore the social impact of the COVID-19 pandemic on young people; (2) examine the extent to which young people are implementing COVID-19 public health guidance; (3) consider the acceptability of vaccination against COVID-19 among young people.”	(1) “Acceptability of vaccination against COVID-19.”
	**Participants’ characteristics**			
	Adolescents aged 12–17 years-old *N = 21*			
(12) Engaging Latino families about COVID-19 vaccines: a qualitative study conducted in Oregon, USA, Garcia et al. (2021)	**USA** *July 2020 to January 2021*	Qualitative *Semi-structured telephone interviews*	“To explore vaccine perceptions among Latino mothers and youth to inform culturally centered strategies that engage the Latino family as a unit for disseminating accurate and culturally appropriate information about vaccines.”	(1) “Tempered optimism for vaccines to protect family well-being”; (2) “Vaccine hesitancy rooted in mistrust of medical and political institutions”; (3) “Intergenerational communication and informational support”; (4) “Meaningful community engagement to convey trustworthy information”
	**Participants’ characteristics**			
	13–18 years-old, in Grades 9 to 12, self-identified as Latinx/ Latino/ Hispanic, and to be able to speak, read, and understand English *N = 24*			
(13) To be or not to be vaccinated against COVID-19 – The adolescents’ perspective – a mixed-methods study in Sweden, Nilsson et al. (2021)	**Sweden** *July to November 2020*	Mixed-methods *Survey*	“The aim of this study was to explore Swedish adolescents’ willingness to be vaccinated against COVID-19 and its association with sociodemographic and other possible factors.”	(1) “The adolescents expressed a need to know more”; (2) “The adolescents did not consider themselves to be in need of vaccination”; (3) “The adolescents expressed a willingness to be vaccinated for the sake of others”
	**Participants’ characteristics**			
	Adolescents aged 15–19 years-old *N = 702*			

### Findings from thematic synthesis

After data extraction and thematic synthesis, five overarching themes were generated: (1) Limited vaccine literacy influences adolescents’ attitudes towards COVID-19 vaccines; (2) Family, peers, and community strongly influence adolescents’ COVID-19 vaccine decision-making; (3) Different levels of trust in vaccine providers and governments influence adolescents’ attitudes towards COVID-19 vaccines; (4) Desire to go back to normality influences adolescents’ COVID-19 vaccine attitudes towards vaccine acceptancy; (5) Autonomy influences adolescents’ COVID-19 vaccine decision-making. These themes and their related categories are summarized in Appendix 3, with exemplifying quotes.

#### Theme 1: Limited vaccine literacy influences adolescents’ attitudes towards COVID-19 vaccines

This theme explores how adolescents’ vaccine literacy further influences their COVID-19 vaccine decisions. Some contradictions regarding vaccination attitudes were present in the studies, however, most studies associated low vaccine literacy with hesitancy or refusal, which was largely represented by low vaccine confidence (55). Low confidence was expressed by many adolescents through concerns about vaccine safety, citing fears of pain from vaccination, needles, unknown long-term side effects, and potential health impacts. Misinformation, mostly through social media platforms, further influences vaccine mistrust through peer criticism or conspiracy beliefs. Rapid vaccine development fuelled these doubts, as reflected in statements like: “*Madam, I do not think it is really reliable because they found the vaccine already in one year … there is also a discussion that there may be other substances in the vaccine so that the world population cannot increase or that women cannot get pregnant– Female Belgian adolescent*” (50). Associated with misinformation, some adolescents from marginalized communities acknowledged that standard public health messaging did not reflect their concerns or realities. Thus, they expressed a need for more relatable, tailored public health messages that resonated with their unique experiences and backgrounds.

Conversely, low vaccine literacy sometimes maintained high confidence, inspired by optimism about vaccine benefits (55, 56), e.g., “[…] *I think it will help and **cure** a lot of people who had COVID-19, and people will be happy to have the vaccine*” (55).

#### Theme 2: Family, peers, and community strongly influence adolescents’ COVID-19 vaccine decision-making

The role of family, peers, and community surfaced as a powerful influence on adolescents’ decision-making. Depending on the degree of trust and proximity, many adolescents tended to adopt the attitudes of trusted family members, which seemed to be particularly strong if these members were vaccine-acceptant HCWs, as exemplified in the quote below: “*Me seeing…my grandmother [changed my mind about the vaccine], she works at a nursing home. She was around numerous patients who had COVID. She was in contact with them and everything, but by her havin’ two of the vaccine shots, she had not caught COVID yet – 15-year-old female living in rural Alabama*” (51). In this case, acceptability was driven by trust in the family member and health outcome after vaccination. Similarly, community norms also significantly influenced attitudes: in China, collective community endorsement of vaccination motivated acceptance, while in other areas, such as parts of Malawi and the DRC, the absence of visible vaccination role models fuelled hesitancy, as reflected in the following statement: “*If I’m told to take the vaccine, I will not take it because… I have not seen anyone get vaccinated, and I would not like to be the first one to be vaccinated”* (50).

#### Theme 3: Different levels of trust in vaccine providers and governments influence adolescents’ attitudes towards COVID-19 vaccines

Adolescents’ confidence in COVID-19 vaccines is strongly shaped by the degree of trust in government and health institutions. Mistrust, particularly among marginalized groups, drives hesitancy, as explained from a rural Black youth in the US, *“…[my friends] just do not trust the government or the systems, and the fact that they think that [the vaccine is] gonna be chipped”* (51). 2SLGBTQ + (Two-Spirit, lesbian, gay, bisexual, transgender, queer (or questioning)) youth also voiced mistrust, often rooted in negative healthcare experiences (49). However, some indicated that trusted HCWs – particularly those with positive connections to the adolescents—could improve vaccine acceptance, as one adolescent explained, *“Trust the advice of medical professionals because they know more than you do”* (56).

#### Theme 4: Desire to go back to normality influences adolescents’ COVID-19 vaccine attitudes towards vaccine acceptance

Adolescents expressed a strong wish to regain a sense of “pre-pandemic normalcy,” motivated by both personal desires and altruistic concerns. Individual reasons for getting vaccinated were often cited, such as “*I want to do a sport and it takes your two doses…for sure I’m going to do it because it’s my sport and well I love it*” (48). Social mandates and vaccine passports further incentivised adolescents to get vaccinated, seeing it as a way to engage in social activities (48). These motivations were often linked more to individualist needs than community protection.

At the same time, however, many did see vaccination as a way to protect and bring stability to their families and communities. For example, one adolescent remarked, *“When you are in a position when it [COVID-19] does not affect you, it would be selfish not to think of other people who you might pass it on to who it would affect”* (52). Many also expressed empathetic attitudes in relation to older adult’s wellbeing, as in “*I think a vaccine against COVID-19 is good because it protects us from getting sick, which leads to society being able to open up and older people do not need to live as isolated as they are now, barely able to go out and with lots of restrictions they have to follow so they do not risk getting sick*” (46). Additional motivations were related to family welfare and opportunities to return to school (57).

#### Theme 5: Autonomy influences adolescents’ COVID-19 vaccine decision-making

Across the literature, adolescents consistently expressed a desire for autonomy in making their own vaccination decisions, both in relation to public health policies and parental authority. In Balma’s research, homeless adolescents experienced their autonomy as limited by institutional policies in health and housing settings (54). Similarly, adolescents from an ethnically diverse underserved community in Canada reported: “It’s like really shitty, sorry, but it’s really shitty because I think it’s really unfair, because it’s your body, your choice, and if you do not want to get the vaccine, that’s your choice, and I do not understand why you should lose your everyday privileges just because you did not get one dose or two” (48). Furthermore, adolescents experienced varying autonomy levels regarding parental influence. In the case of HCW parents, a physician’s child felt compelled to state*, “My dad was probably gonna make me do it… I’d agreed with him*,” while a nurse’s child felt fully autonomous, saying, “*I would probably put it at 100% my own decision”* (56). In some instances, adolescents even challenged their family members’ opinions, as in the following example in a Brazilian context: “*Then she [grandmother] thinks she is not going to get the vaccine, and I say ‘where is your sister to take care of you?’ Because her sister keeps putting in her head that this vaccine is from the devil, that it’s this, that it’s that. Then I said, ‘well, now you call your sister to take care of you, because you do not listen to us*” (50). This illustrates the adolescents’ ability to self-govern and act according to their own values.

### Quality assessment

The quality of each included study was assessed using the JBI Critical Appraisal Checklist for Qualitative Research, as summarized in Appendix 2. Among the thirteen studies, nine were rated as medium methodological quality (46, 47, 49–52, 54, 56, 58), three as high quality (48, 55, 57) and one as low quality (53). However, none were excluded due to quality. Notably, eleven studies (46, 47, 49–54, 56–58) did not position the researcher culturally or theoretically, nor did they acknowledge the reciprocal influence between the researcher and the research. Additionally, Balma et al. cited that they did not require IRB ethical approval, but did obtain consent when conducting the interviews (54).

## Discussion

This study systematically reviewed qualitative research to explore factors influencing COVID-19 vaccine decision-making and hesitancy among adolescents. Thirteen articles were analysed, revealing five key themes about the role of vaccine literacy, the influence of personal relationships, the impact of levels of trust, the desire to return to normalcy, and the adolescents’ feelings of autonomy on vaccine decision making.

First, our study illustrated the complex relationship between vaccine literacy and vaccine decision-making. One finding that there is a direct relationship between low literacy and low confidence aligns with existing literature and often arises from a lack of understanding, insufficient information, or spread of misinformation (59, 60). However, we also found that the inverse could be true. A study suggested that vaccine-literate people can also refuse vaccines due to not knowing how to interpret received messages from an overload of information (61). Similarly, so-called “vaccine enquirers” refers to individuals with high vaccine literacy, who after receiving informed research make deliberate vaccination choices within the continuum of vaccination attitudes. This distinction emphasizes their confidence and agency in decision-making, suggesting that public health narratives could shift to avoid vaccine-related stigma by recognizing this empowered approach (62). However, we also observed an inverse relationship between low vaccine literacy and high confidence, which might be attributable more to external influences than low knowledge itself. Thus, the relationship between vaccine literacy and adolescent vaccine decision-making is not always straightforward.

To address barriers relating to vaccine literacy and misinformation, the adolescents voiced a need for tailored vaccine messaging. The typical information received was often not well understood or not clearly provided (13, 63). Particularly among adolescents in vulnerable situations, there was a sense that existing vaccine messages did not align with their experiences or livelihood. This suggests that improving vaccine messaging clarity and accessibility could enhance vaccine literacy and foster informed decision-making. Additionally, it is crucial to ensure that credible, trusted sources are accessible to adolescents to counter false narratives effectively.

Notably, in our study, the low levels of vaccine confidence in vulnerable groups extend beyond information and seemed to be deeply rooted in government or health systems mistrust. This mistrust often stemmed from a history of marginalization and experience of mistreatment by health systems and governments. This finding was also present in systematic reviews of vaccine hesitancy among vulnerable groups (64), the general population (16, 17, 65) and during pandemics (66). Addressing these deep-seated structural inequalities and general mistrust could help replace hesitant attitudes with greater confidence. One potential solution is to harness the existing trust in healthcare workers who are considered trustworthy providers of both vaccines and information. Other common and trusted sources of information include the community and family, who could also be avenues for improving adolescent vaccine confidence.

Confidence also includes trust in the effectiveness and safety of the vaccines themselves. Fears around safety and long-term effects of COVID-19 vaccines were highlighted by adolescents in this study. This anxiety about long-term consequences might reflect broader vaccine hesitancy trends, particularly in relation to newer vaccines, as low confidence is not prevalent in all vaccine types (67). These concerns are often driven by factors such as misinformation and a lack of institutional trust. Additionally, fear surrounding more immediate concerns of vaccination, such as the perceived pain associated with the injection and fear of needles, were also consistently reported in other studies (18, 67). This highlights the need for strategies that not only build trust in vaccine safety but also take into consideration procedural fears, especially among younger populations.

This review, similar to prior evidence, also identified complacency in vaccination relating to low perceived risk for severe illness among adolescents (66, 67). It should be noted though, that in other diseases, such as hepatitis B, low perceived risk seemed to reflect the adolescent’s lack of understanding of disease transmission (67). This low perceived risk may stem from misinformation and a lack of trust in public health authorities among disadvantaged groups, while in more advantaged groups, it may reflect a sense of superiority toward public health agencies (68). Contrary to the cited complacency, other studies in the review showed that a vaccination driver is an altruistic desire to protect others, such as their family or the community. In addition to that, the desire to go back to a sense of pre-pandemic normalcy, influenced by both individualistic and collective motives, was another motivation for vaccination. This desire was found to be common among the general population during other pandemic studies (66, 69). Therefore, both individual risk perceptions and collective motivations may be key to addressing vaccine-hesitant attitudes.

Finally, at an individual level, adolescents showcased a desire to make autonomous health-related choices, echoing findings of previous general vaccine studies (18, 67). However, their autonomy was often limited by local legislation and parental authority, which frequently overruled adolescents’ choices, particularly in certain contexts. An additional barrier might also be HCWs’ hesitancy to vaccinate adolescents without parental approval, even in situations where legislation allowed for self-consent (67). As a result, factors such as legislation, parental authority, HCWs’ practices, and broader contextual influences all played a significant role in shaping adolescents’ autonomy in vaccine decision-making.

It is essential to recognize that all these factors must be considered within the context of different settings and the diverse socioeconomic backgrounds of adolescents. Context plays a critical role in shaping perceptions, as it is closely linked to specific policies and mandates, levels of institutional trust, and the degree of autonomy individuals have in making health decisions. Socioeconomic factors such as education and income can significantly impact access to vaccination services and information, while cultural beliefs further shape attitudes toward vaccination (18). Additionally, individual perception of health status, particularly in the presence of pre-existing conditions, influences vaccine perceptions, often affecting the perceived risks and benefits of immunization (70). Given the complexity of these factors, future research should explore adolescent attitudes toward vaccination across various sociocultural and economic contexts. Comparative studies across different regions and policy environments could provide valuable insights into how institutional trust, autonomy, and socioeconomic disparities shape vaccine decision-making.

Institutional trust emerged as a key factor influencing nearly all dimensions of vaccination decision-making among adolescents. Therefore, policymakers should not only ensure that vaccine-related information is accessible and relevant to adolescents but also foster open and transparent dialogue to build trust in government institutions and healthcare professionals (50). Empowering adolescents to make informed decisions about their health, will not only enhance trust but also contribute to a more resilient and health-conscious generation.

### Limitations

This study had several limitations that should be considered. Firstly, the included studies had varying aims, research questions, data collection instruments, and interview guides, introducing heterogeneity in the findings. While this diversity may make it challenging to draw entirely consistent conclusions, it also provides a more comprehensive understanding of the topic by capturing different perspectives and offering a richer, more nuanced picture of vaccine uptake. Moreover, weaker or moderate-quality studies were included, potentially skewing results and compromising reliability. However, given our objective to capture all relevant insights, we chose to include these studies while acknowledging their quality. This approach ensured that we did not overlook valuable perspectives that could contribute to a broader understanding of adolescent vaccine hesitancy. Another key limitation was the variability in contextual factors, as vaccine availability varied across different countries and the timing of the studies. More favourable findings regarding vaccine uptake may have been observed in regions where vaccines were readily available, alongside visible reductions in COVID-19-related deaths. Additionally, local laws, cultural norms, and societal contexts differ between countries, limiting the generalizability of our results across settings. Nonetheless, our aim was to provide a broad synthesis of the evidence, and by including studies from diverse contexts, we ensured that the review captured a wide range of experiences and decision-making processes, making the findings more globally relevant.

## Conclusion

This systematic review highlights the complex and interconnected factors influencing COVID-19 vaccine decision-making among adolescents. The findings demonstrate that adolescents’ vaccine confidence is multifaceted, shaped not only by their own understanding of vaccines but also by the attitudes of trusted social networks and institutions. Misinformation and mistrust, particularly among vulnerable groups, continue to be significant barriers to vaccine acceptance. In contrast, trusted HCWs and family and community members, along with the longing for a return to normal life, were crucial in promoting positive vaccination attitudes. In addition, autonomy was found to be important for adolescents. Therefore, these findings suggest that enhancing vaccine literacy through tailored, accessible messaging, addressing structural mistrust, and empowering adolescents to make autonomous health decisions are critical for improving vaccine acceptance. Future public health strategies should consider these nuanced influences in diverse contexts to effectively engage adolescents.

## Data Availability

The original contributions presented in the study are included in the article/Supplementary material, further inquiries can be directed to the corresponding author.
