# Supplementary material for: Factors influencing adolescents’ decision-making about COVID-19 vaccination: a systematic review with qualitative synthesis
Source: Front Public Health. 2025 May 14;13:1563677. doi: 10.3389/fpubh.2025.1563677 (PMC12116342; doi:10.3389/fpubh.2025.1563677)
Supplement: Supplementary file 1 [file Supplementary_file_1.docx]

Appendix 1. Full search strategy for databases

| 1. Sociological abstracts | | | | |
| --- | --- | --- | --- | --- |
| Interface: ProQuest  Date of Search: 23 February 2024  Number of hits: 59  Comment: Includes companion file Social Services Abstracts | Field labels   - noft = anywhere except full text - ti,ab,if = title, abstract, keyword - MAINSUBJECT.EXACT = non exploded subject heading - MAINSUBJECT.EXACT.EXPLODE = exploded subject heading - NEAR/x = within x words, regardless of order - * = truncation of word for alternate endings   Note: sometimes “quotation marks” are needed for single search terms to avoid automatic term mapping (lemmatization). | | | |
| \| S1 \| MAINSUBJECT.EXACT("Vaccination") OR (TI,AB,IF(immunizat* OR immunisat OR nonvaccin* OR non-immun* OR nonimmun* OR unimmun* OR un-immun* OR unvaccin* OR vaccin*)) \| 3,516 \| \| --- \| --- \| --- \| \| S2 \| MAINSUBJECT.EXACT("Awareness") OR MAINSUBJECT.EXACT("Consciousness") OR MAINSUBJECT.EXACT("Choices") OR (MAINSUBJECT.EXACT("Medical Decision Making") OR MAINSUBJECT.EXACT("Decision Making")) OR MAINSUBJECT.EXACT("Fear") OR MAINSUBJECT.EXACT("Intentionality") OR MAINSUBJECT.EXACT("Trust") OR ((immunizat* OR immunisat* OR vaccin*) NEAR/3 (confiden* OR delay* OR hesitan* OR refuse OR refuses OR refusing OR refusal* )) OR (TI,AB,IF(anti-vaccin* OR antivaccin* OR anti-vax* OR antivax* )) OR (TI,AB,IF((immunizat* OR immunisat* OR vaccin* ) AND (accept* OR attitude* OR awareness OR barrier* OR behavior* OR behaviour* OR belief* OR choice* OR compulsory OR concern* OR conscious* OR controvers* OR critic* OR decision-make* OR decision-making* OR dilemma* OR distrust OR doubt* OR dropout* OR enable* OR exemption* OR fear* OR intent* OR knowledge OR mandatory OR misconception* OR misinformat* OR mistrust* OR objection* OR objector* OR opposition* OR perception* OR reject* OR reluctan* OR rumor* OR rumour* OR trust* OR uptake* OR willing* OR unconscious* OR unwilling* ))) \| 66,690 \| \| S3 \| MAINSUBJECT.EXACT("Adolescents") OR MAINSUBJECT.EXACT("Young Adults") OR (MAINSUBJECT.EXACT("College Students") OR MAINSUBJECT.EXACT("Junior High School Students") OR MAINSUBJECT.EXACT("Students") OR MAINSUBJECT.EXACT("Foreign Students") OR MAINSUBJECT.EXACT("High School Students")) OR (TI,AB,IF (adolescen* OR juvenile* OR minor OR minors OR student* OR teen OR teens OR teenager* OR young OR younger OR youth )) \| 135,313 \| \| S4 \| S1 AND S2 AND S3 \| 159 \| \| S5 \| (S1 AND S2 AND S3) AND pd(20000101-20230705) \| 59 \| | | | | |
| 2. Web of Science Core Collection | | | | |
| Interface: Clarivate Analytics  Editions = A&HCI , ESCI , SCI-EXPANDED , SSCI  Date of Search: 23 February 2024  Number of hits: 2,067 | | | Field labels   - TS/Topic = title, abstract, author keywords and Keywords Plus - NEAR/x = within x words, regardless of order - * = truncation of word for alternate endings   Note: the *Exact search*-function was used for all the searches | |
| \| # \| Search Query \| Results \| \| --- \| --- \| --- \| \| 1 \| TS=(immuni$at* OR nonvaccin* OR non-immun* OR nonimmun* OR unimmun* OR un-immun* OR unvaccin* OR vaccin* ) \| 541168 \| \| 2 \| TS=(anti-vaccin* OR antivaccin* OR anti-vax* OR antivax* ) \| 1522 \| \| 3 \| TS=((vaccin* OR immuni$at* ) NEAR/2 (confiden* OR delay* OR hesitan* OR refuse$ OR refusing OR refusal* )) \| 11114 \| \| 4 \| AB=((adolescen* OR boy$ OR girl$ OR juvenile* OR minor OR minors OR student* OR teen OR teens OR teenager* OR young OR younger OR youth ) AND ((immuni$at* OR vaccin* ) NEAR/2 (accept* OR attitude* OR awareness OR barrier* OR behavio$r* OR belief* OR choice* OR compulsory OR concern* OR conscious* OR controvers* OR critic* OR decision-make* OR decision-making* OR dilemma* OR distrust OR doubt* OR dropout* OR enable* OR exemption* OR fear* OR intent* OR knowledge OR mandatory OR misconception* OR misinformat* OR mistrust* OR objection* OR objector* OR opposition* OR perception* OR reject* OR reluctan* OR rumo$r* OR trust* OR uptake* OR willing* OR unconscious* OR unwilling* ))) \| 5626 \| \| 5 \| TI=((immuni$at* OR vaccin* ) NEAR/2 (accept* OR attitude* OR awareness OR barrier* OR behavio$r* OR belief* OR choice* OR compulsory OR concern* OR conscious* OR controvers* OR critic* OR decision-make* OR decision-making* OR dilemma* OR distrust OR doubt* OR dropout* OR enable* OR exemption* OR fear* OR intent* OR knowledge OR mandatory OR misconception* OR misinformat* OR mistrust* OR objection* OR objector* OR opposition* OR perception* OR reject* OR reluctan* OR rumo$r* OR trust* OR uptake* OR willing* OR unconscious* OR unwilling* )) OR AK=((immuni$at* OR vaccin* ) NEAR/2 (accept* OR attitude* OR awareness OR barrier* OR behavio$r* OR belief* OR choice* OR compulsory OR concern* OR conscious* OR controvers* OR critic* OR decision-make* OR decision-making* OR dilemma* OR distrust OR doubt* OR dropout* OR enable* OR exemption* OR fear* OR intent* OR knowledge OR mandatory OR misconception* OR misinformat* OR mistrust* OR objection* OR objector* OR opposition* OR perception* OR reject* OR reluctan* OR rumo$r* OR trust* OR uptake* OR willing* OR unconscious* OR unwilling* )) \| 11164 \| \| 6 \| #2 OR #3 OR #4 OR #5 \| 23111 \| \| 7 \| #1 AND #6 \| 23003 \| \| 8 \| AB=((vaccin* OR immuni$at* ) NEAR/2 (adolescen* OR boy$ OR girl$ OR juvenile* OR minor OR minors OR student* OR teen OR teens OR teenager* OR young OR younger OR youth )) \| 6649 \| \| 9 \| TI=(adolescen* OR boy$ OR girl$ OR juvenile* OR minor OR minors OR student* OR teen OR teens OR teenager* OR young OR younger OR youth ) OR AK=(adolescen* OR boy$ OR girl$ OR juvenile* OR minor OR minors OR student* OR teen OR teens OR teenager* OR young OR younger OR youth ) \| 1219824 \| \| 10 \| #8 OR #9 \| 1223546 \| \| 11 \| #7 AND #10 \| 4036 \| \| 12 \| #11 Timespan: 2020-01-01 to 2024-12-31 \| 2067 \| | | | | |
| 3. Medline | | | | |
| Interface: Ovid MEDLINE(R) ALL  Date of Search: 23 February 2024  Number of hits: 3,326  Comment: In Ovid, two or more words are automatically searched as phrases; i.e. no quotation marks are needed  The Ovid MEDLINE®️ database contains records with the following possible status besides MEDLINE: Publisher, In-Data-Review, In-Process and PubMed-not-MEDLINE records from NLM. | | | | Field labels   - exp/ = exploded MeSH term - / = non exploded MeSH term - .ti,ab,kf. = title, abstract and author keywords - adjx = within x words, regardless of order - * = truncation of word for alternate endings |
| Database(s): Ovid MEDLINE(R) ALL 1946 to February 22, 2024 Search Strategy:   \| **#** \| **Searches** \| **Results** \| \| --- \| --- \| --- \| \| 1 \| Immunization/ \| 54043 \| \| 2 \| Immunization Programs/ \| 13004 \| \| 3 \| exp Vaccination/ \| 113134 \| \| 4 \| exp COVID-19 Vaccines/ or Vaccines/ or Viral Vaccines/ \| 80415 \| \| 5 \| (immuni?at* or nonvaccin* or non-immun* or nonimmun* or unimmun* or un-immun* or unvaccin* or vaccin*).ti,ab,kf. \| 513010 \| \| 6 \| or/1-5 \| 554321 \| \| 7 \| Anti-Vaccination Movement/ \| 182 \| \| 8 \| Patient Acceptance of Health Care/ \| 55570 \| \| 9 \| exp Vaccination Refusal/ \| 1841 \| \| 10 \| (anti-vaccin* or antivaccin* or anti-vax* or antivax*).ti,ab,kf. \| 1316 \| \| 11 \| ((vaccin* or immuni?at*) adj3 (confiden* or delay* or hesitan* or refuse? or refusing or refusal*)).ti,ab,kf. \| 10937 \| \| 12 \| Awareness/ \| 22207 \| \| 13 \| Behavior/ \| 30210 \| \| 14 \| Choice Behavior/ \| 35123 \| \| 15 \| Communication Barriers/ \| 7343 \| \| 16 \| Consciousness/ \| 13964 \| \| 17 \| Decision Making/ \| 105596 \| \| 18 \| Fear/ \| 39551 \| \| 19 \| Health Knowledge, Attitudes, Practice/ \| 127906 \| \| 20 \| Intention/ \| 17164 \| \| 21 \| exp Mandatory Programs/ \| 7208 \| \| 22 \| Trust/ \| 13438 \| \| 23 \| ((adolescen* or boy? or girl? or juvenile* or minor or minors or student* or teen or teens or teenager* or young or younger or youth) and ((immuni?at* or vaccin*) adj3 (accept* or attitude* or awareness or barrier* or behavio?r* or belief* or choice* or compulsory or concern* or conscious* or controvers* or critic* or decision-make* or decision-making* or dilemma* or distrust or doubt* or dropout* or enable* or exemption* or fear* or intent* or knowledge or mandatory or misconception* or misinformat* or mistrust* or objection* or objector* or opposition* or perception* or reject* or reluctan* or rumo?r* or trust* or uptake* or willing* or unconscious* or unwilling*))).ab. \| 6105 \| \| 24 \| ((immuni?at* or vaccin*) adj3 (accept* or attitude* or awareness or barrier* or behavio?r* or belief* or choice* or compulsory or concern* or conscious* or controvers* or critic* or decision-make* or decision-making* or dilemma* or distrust or doubt* or dropout* or enable* or exemption* or fear* or intent* or knowledge or mandatory or misconception* or misinformat* or mistrust* or objection* or objector* or opposition* or perception* or reject* or reluctan* or rumo?r* or trust* or uptake* or willing* or unconscious* or unwilling*)).ti,kf. \| 10054 \| \| 25 \| exp Vaccination/px \| 2869 \| \| 26 \| or/7-25 \| 454631 \| \| 27 \| 6 and 26 \| 30290 \| \| 28 \| Adolescent/ \| 2236154 \| \| 29 \| exp Students/ \| 173451 \| \| 30 \| Young Adult/ \| 1022107 \| \| 31 \| ((vaccin* or immuni?at*) adj3 (adolescen* or boy? or girl? or juvenile* or minor or minors or student* or teen or teens or teenager* or young or younger or youth)).ab. \| 7721 \| \| 32 \| (adolescen* or boy? or girl? or juvenile* or minor or minors or student* or teen or teens or teenager* or young or younger or youth).ti,kf. \| 718963 \| \| 33 \| or/28-32 \| 3118397 \| \| 34 \| 27 and 33 \| 9338 \| \| 35 \| limit 34 to yr="2020 -Current" \| 3369 \| \| 36 \| limit 35 to english language \| 3326 \| | | | | |
| 4. Publicly Available Content Database | | | | |
| Interface: ProQuest  Date of Search: 23 February 2024  Number of hits: 1,123 | | Field labels   - noft = anywhere except full text - ti,ab,if = title, abstract, keyword - NEAR/x = within x words, regardless of order - * = truncation of word for alternate endings   Note: sometimes “quotation marks” are needed for single search terms to avoid automatic term mapping (lemmatization). | | |
| \| S1 \| TI,AB,IF(immunisat* OR immunizat* OR nonvaccin* OR non-immun* OR nonimmun* OR unimmun* OR un-immun* OR unvaccin* OR vaccin*) \| 80,249 \| \| --- \| --- \| --- \| \| S2 \| TI,AB,IF(anti-vaccin* OR antivaccin* OR anti-vax* OR antivax*) \| 447 \| \| S3 \| (TI,AB,IF((vaccin* OR immuni?at*) NEAR/3 (confiden* OR delay* OR hesitan* OR refuse? OR refusing OR refusal*))) \| 3,586 \| \| S4 \| (TI,AB,IF((immunizat* OR immunisat* OR vaccin* ) AND (accept* OR attitude* OR "awareness" OR barrier* OR behavior* OR behaviour* OR belief* OR choice* OR "compulsory" OR concern* OR conscious* OR controvers* OR critic* OR decision-make* OR decision-making* OR dilemma* OR "distrust" OR doubt* OR dropout* OR enable* OR exemption* OR fear* OR intent* OR "knowledge" OR "mandatory" OR misconception* OR misinformat* OR mistrust* OR objection* OR objector* OR opposition* OR perception* OR reject* OR reluctan* OR rumor* OR rumour* OR trust* OR uptake* OR willing* OR unconscious* OR unwilling* ))) \| 27,945 \| \| S5 \| [S2] OR [S3] OR [S4] \| 28,553 \| \| S6 \| [S1] AND [S5] \| 28,539 \| \| S7 \| (TI,IF(adolescen* OR juvenile* OR "minor" OR "minors" OR student* OR "teen" OR "teens" OR teenager* OR young OR younger OR youth )) \| 178,315 \| \| S8 \| (AB((vaccin* OR immuni?at*) NEAR/3 (adolescen* OR juvenile* OR "minor" OR "minors" OR student* OR "teen" OR "teens" OR teenager* OR "young" OR "younger" OR "youth"))) \| 1,538 \| \| S9 \| [S7] OR [S8] \| 179,063 \| \| S10 \| [S6] AND [S9] \| 1,648 \| \| S11 \| ([S6] AND [S9]) AND pd(20000101-20230705) \| 1,123 \| | | | | |
