# Supplementary material for: Factors influencing adolescents’ decision-making about COVID-19 vaccination: a systematic review with qualitative synthesis
Source: Front Public Health. 2025 May 14;13:1563677. doi: 10.3389/fpubh.2025.1563677 (PMC12116342; doi:10.3389/fpubh.2025.1563677)
Supplement: Supplementary file 2 [file Supplementary_file_2.docx]

Appendix 2: JBI Critical Appraisal Checklist For Qualitative Research

| study | Q1 | Q2 | Q3 | Q4 | Q5 | Q6 | Q7 | Q8 | Q9 | Q10 | Overall Appraisal |
| --- | --- | --- | --- | --- | --- | --- | --- | --- | --- | --- | --- |
| 1) Groenewald, C, et al. (56) | Y | Y | Y | Y | Y | Y | Y | Y | Y | Y | A (strong) |
| 2) Persaud, Y., et al. (59) | Y | Y | Y | Y | Y | N | N | Y | Y | Y | B (medium) |
| 3) Balma, B., et al. (55) | Y | Y | Y | Y | Y | U | U | Y | N | Y | B (medium) |
| 4) Alemu, D., et al.(54) | U | U | U | U | N | N | Y | N | Y | Y | C (weak) |
| 5) Budhwani, H., et al. (40) | Y | Y | Y | Y | Y | N | N | Y | Y | Y | B (medium) |
| 6) Mansfield, L. N., et al. (57) | Y | Y | Y | Y | Y | N | U | Y | Y | Y | B (medium) |
| 7) Garcia, J., et al. (58) | Y | Y | Y | Y | Y | N | Y | Y | Y | Y | A (strong) |
| 8) Abramovich, A., et al. (50) | Y | Y | Y | Y | Y | N | N | Y | Y | Y | B (medium) |
| 9) Fisher, H., et al. (53) | Y | Y | Y | Y | Y | N | N | Y | U | Y | B (medium) |
| 10) Ramaiya, A., et al. (51) | Y | Y | Y | Y | Y | N | N | Y | Y | Y | B (medium) |
| 11) Logie, C. H., et al. (48) | Y | Y | Y | Y | Y | U | U | Y | Y | Y | B (medium) |
| 12) McKinnon, B., et al. (49) | Y | Y | Y | Y | Y | Y | Y | Y | Y | Y | A (strong) |
| 13) Nilsson, S., et al. (47) | Y | Y | Y | Y | Y | U | U | Y | Y | Y | B (medium) |
| JBI – Joanna Briggs Institute  Y = yes. Indicates clear statement in the paper that directly answers the question.  N = no. Indicates the question has not been addressed by the paper.  U = unclear. No clear or ambiguous information presented in the paper.  Questions:  Q1: is there congruity between the stated philosophical perspective and the research methodology?  Q2: is there congruity between the research methodology and the research question or objectives?  Q3: is there congruity between the research methodology and the methods used to collect data?  Q4: is there congruity between the research methodology and the representation and analysis of data?  Q5: is there congruity between the research methodology and the interpretation of results?  Q6: is there a statement locating the researcher culturally or theoretically?  Q7: is the influence of the researcher on the research, and vice- versa, addressed?  Q8: are participants, and their voices, adequately represented?  Q9: is the research ethical according to current criteria or, for recent studies, and is there evidence of ethical approval by an appropriate body?  Q10: do the conclusions drawn in the research report flow from the analysis, or interpretation, of the data? | | | | | | | | | | | |
