# Supplementary material for: Factors influencing adolescents’ decision-making about COVID-19 vaccination: a systematic review with qualitative synthesis
Source: Front Public Health. 2025 May 14;13:1563677. doi: 10.3389/fpubh.2025.1563677 (PMC12116342; doi:10.3389/fpubh.2025.1563677)
Supplement: Supplementary file 3 [file Supplementary_file_3.docx]

Appendix 3: Overarching themes, categories and studies excerpts derived from thematic synthesis.

| **Theme 1: Limited vaccine literacy influences adolescents´ attitudes towards COVID-19 vaccines** |
| --- |
| Category 1.1: Low vaccine literacy associated with exposure to misinformation (*infodemic*, fake news, conspiracy theories, anecdotal narratives) leads to vaccine mistrust |
| - “As one youth noted, misinformation and lack of reputable sources was a problematic influence for young people: ‘Unless they cite their sources, unfortunately. It’s just–people can say whatever they want and people believe them, unfortunately. And it’s a problem’” (55) - “I have seen information about the COVID-19 vaccine from Facebook that it has severe side effects.” (54) - “[…] there are people who believe that Elon Musk, I think, who plants a GPS on us. If you’re vaccinated, he’s going to track you, he’s going to know everything about you, or they’re going to put viruses on you, in your body, and you’ll go to the hospital and waste money. Not here in Canada, but like more so in the U.S. Yeah that’s stuff I’ve heard”. (49) |
| Category 1.2: Lack of knowledge about vaccine development process affects vaccine confidence |
| - “I am concerned that this vaccine had become available within one year but on the other we do not have a vaccine for HIV, for TB, and for SIT. . . so, it has taken about 22 years without vaccine for those but the one for COVID-19 they were able to get it sooner? So, there is something scary about this vaccine for COVID. Perhaps they developed this vaccine for COVID, but they are not sure what will actually happen in a year or two”. (56) - “And why are there mRNAs in there, as well? Which is a very big concern why you have modified DNA ingredients in there. Very, very big concern”. (55) |
| Category 1.3: Vaccine confident adolescents have low knowledge about COVID vaccines |
| - “I think it (COVID-19 vaccines] will help many people like those with weak immune systems; like the elderly and the kids”. She further added that she is excited about the vaccines because “people will be treated for COVID-19”. (56) |
| Category 1.4: Low vaccine literacy affects concerns or fears of long-term effects of vaccines |
| - “Yes, what if it does not work? What if it protects one for just a certain time?” (56) - “What’s the long-term effects that it’s gonna have on my body futuristics?” (55) - “I would get vaccinated if proper tests have been done – so it’s not like that influenza vaccine that could bring on narcolepsy”. (47) |
| Category 1.5: Low vaccine literacy affects concerns or fears of side-effects or short-term effects related to vaccination |
| - “I don’t know the risks it could have on me, I would like to know the pros and cons about the vaccination”. (59) - “Youth were concerned about side effects for various reasons, including not wanting to be sick or being unable to fulfill daily responsibilities”. (50) - “I have a needle phobia and I’m scared of the bad sides of a vaccine that haven’t been found yet but that people discover after the vaccination has been done”. (47) |
| Category 1.6: Vaccine messaging and outreach need improvement |
| - “YEH commented that trusted vaccine information should appear on mass transit, a space where agency information is frequently displayed”. (55) - “To dispel mistrust, participants suggested informational campaigns that target venues with cultural significance, feature trusted community advocates, and acknowledge historical trauma”. (58) |
| **Theme 2: Family, peers, and community strongly influence adolescents´ attitudes towards COVID-19 vaccines** |
| Category 2.1: Close and trusted family members play an important role in adolescents´ COVID-19 vaccine decision-making |
| - “In family PS2, both parent and child linked COVID-19 vaccines to something that is taken only when someone has been infected with COVID-19”. (56) - “Most were told about the vaccines by their parents or caregivers and the majority were willing to receive a COVID-19 vaccine, reflecting a great level of vaccine acceptability in the sample”. (56) - “My family is personally against it, being a Latina female. So, they wouldn’t take it.” (55) |
| Category 2.2: Adolescents´ decision-making process is influenced by vaccine related information deriving from or influenced by the community/ societal norms |
| - “It (COVID-19 vaccines) will protect us, and I heard a lot have been vaccinated around (my province). People have to get it!”. (56) - “To improve trust, participants believed that information should be disseminated through well-known Latino leaders, advocacy groups, and community health workers (CHWs)”. (58) |
| Category 2.3: Low perceived risk to get COVID or to become gravely ill |
| - “I will not take it since I am not sick”. (56) - “I’m at low risk of COVID-19 disease and I don’t want to get a vaccine against COVID-19…I don’t trust the COVID-19 vaccine” (54) - “They did not fear COVID-19 as death rates are low in their age group and some said they would rather be infected than vaccinated” (47) |
| Category 2.3: Influence of church or religion |
| - “...my pastor was sayin’, it’s like, y’all should go get this vaccine, so we can be safe up in here, since we got some church members who has taken the vaccine and some who have not. He was totally like, our Black folks in general, do not believe in this COVID, because y’all will not take the vaccine and it might spike again…He was like this vaccine is very important, so when they bring it back, and come back with it, ya’ll should go take it. Keep all us safe. Keep your family and friends safe. The vaccine is good”. (52) - “It is a means to transmit COVID-19 disease and spread the illuminati religion or Satanism…My religion also doesn’t permit you to take this vaccine”. (54) |
| **Theme 3: Different levels of trust in vaccine providers and governments influence adolescents´ attitudes towards COVID-19 vaccines** |
| Category 3.1: The degree of trust in government/policies correlates positively with vaccine confidence |
| - “Yeah. I think the system or the government admits they’re sneaky. They do not really care for the people for real. They just care about money and power”. (52) - “saw the Minister of Health in Uganda was vaccinated so I would also welcome the vaccine” (48) |
| Category 3.2: The degree of trust in healthcare systems/workers correlates positively with vaccine confidence |
| - “Trust the advice of medical professionals because they know more than you do.” (57) - Youth who were vaccine non-confident were less likely to trust doctors, healthcare providers, public health agencies, and government websites. (50) |
| Category 3.3: Adolescents in vulnerable situations or from historically marginalized groups demonstrate higher levels of vaccine mistrust |
| - “They’re lying. You know, some people think it’s a population control attempt. You know, racist. Like, some certain drugs were made by the government to get rid of Black people. It happened.” (55) - “It was also noted that trauma and ongoing mental health issues may have impacted participants’ perceptions of COVID-19 and interest in getting vaccinated, such that the risk of infection may not have been as great of a concern for individuals facing multiple intersections of systemic and societal marginalization.” (50) |
| **Theme 4: Desire to go back to normality influences adolescents´ COVID-19 vaccine attitudes towards vaccine acceptancy** |
| Category 4.1: Adolescents' motivation to get vaccinated is influenced by their desire to protect themselves while engaging in activities beyond their living setting (self-protection) |
| - “I hate needles and getting shots, but I was really excited to go get my vaccine because I knew it meant we were one step closer to getting back to normal.” (57) |
| Category 4.2: Adolescents are motivated to get vaccinated themselves and advocate for vaccination among others, aiming to protect the community from the virus and restore societal health, leading to a return to normalcy |
| - “I think it probably is better for them [older people] to get it just so they know that they’re less in danger and they have more things to do because obviously the lockdown can’t last forever and they’re eventually gonna get older and older so they don’t want to spend their last days staying in their house for 10 or 11 weeks.” (53) |
| Category 4.3: Adolescents´ motivation to be vaccinated is to comply with vaccine mandates so the return to pre-pandemic normality will be possible |
| - “Well if it goes on like this, if, because if you’re not vaccinated, you can’t go eat out, you can’t do much, in fact, you can’t do anything. If it goes on like this, I’ll just have no choice but to take the vaccine, because if I don’t, I don’t want to be locked up in my room for the rest of my life.” (49) |
| **Theme 5: Autonomy influences adolescents´ COVID-19 vaccine decision-making** |
| Category 5.1: Adolescents assertively desire to be autonomous in vaccine decision-making |
| - “I wouldn’t try to force anyone to get the vaccine. I know it’s safe but don’t want anyone to be uncomfortable about doing something they don’t want.” (57) - “Because I think it’s my own freedom, my own right. If I don’t want to take the vaccine, I won’t take it. They can’t force me. They can do whatever they want, I’m not going to take the vaccine.” (49) |
| Category 5.2: Adolescents yield to parental influence in vaccine decision-making |
| - “I didn’t want to take it, but then I changed my mind cause so many people were taking it. Even if I didn’t want to get it, they would still make me get it.” (57) |
| Category 5.3: Adolescents with strong sense of autonomy often criticize family members and community when they’re in opposite sides of the vaccine hesitancy continuum |
| - “Then she [grandmother] thinks she is not going to get the vaccine, and I say "where is your sister to take care of you?” Because her sister keeps putting in her head that this vaccine is from the devil, that it's this, that it's that. Then I said "well, now you call your sister to take care of you, because you don't listen to us". (51) |
